# Evaluation of anxiety, embarrassment, and pain associated with cystoscopy in the operating room: a male population sample

**DOI:** 10.1590/1806-9282.20250888

**Published:** 2025-12-05

**Authors:** Yasemin Özhanlı, Açelya Türkmen, Nadiye Alkay, Duygu Soytaş, Uğur Yücetaş

**Affiliations:** 1 Kocaeli University, Faculty of Health Sciences, Department of Surgical Nursing – İzmit, Türkiye.; 2 Çukurova University, Faculty of Health Sciences, Department of Surgical Diseases Nursing – Adana, Türkiye.; 3 Istanbul University-Cerrahpaşa, Cerrahpaşa Medical Faculty Hospital Nursing Directorate – Istanbul, Türkiye.; 4 Istanbul Training and Research Hospital, Training Unit – Istanbul, Türkiye.; 5 Istanbul Training and Research Hospital, Department of Urology – Istanbul, Türkiye.

**Keywords:** Pain, Embarrassment, Anxiety, Cystoscopy

## Abstract

**OBJECTIVE:**

The aim of this study was to determine the anxiety, embarrassment, and pain levels of patients undergoing a cystoscopy procedure.

**METHODS:**

This cross-sectional study was conducted on 151 male patients who underwent cystoscopy in a hospital operating room between October 2021 and 2022. Data were collected with the Beck Anxiety Inventory for anxiety in preoperative; the Numeric Rating Scale for anxiety, embarrassment, and pain in intraoperative.

**RESULTS:**

The mean score of the patients’ Beck Anxiety Inventory was 3.810±6.905; the mean scores of the first and second Numeric Rating Scale measurements were 1.79±1.23; 1.94±1.28 for anxiety; 2.77±2.54; 2.32±2.30 for embarrassment; 1.44±0.75; 3.46±2.15 for pain, respectively. As a result of the regression analysis, the anxiety levels of the patients before the procedure explained 15.2 and 38.6% of the embarrassment and pain levels during the procedure, respectively.

**CONCLUSION:**

Anxiety, embarrassment, and pain levels of the patients in terms of the cystoscopy procedure were related to each other, and these parameters varied during different steps of the procedure.

## BACKGROUND

Cystoscopy is an important and valuable diagnostic tool in daily urological practice. This procedure is part of the ­evaluation process in patients complaining of lower urinary tract symptoms and can be performed repeatedly in patients being monitored for non-invasive bladder tumors^1^. With very low side effects and morbidity, this procedure allows direct visual inspection of the bladder cavity and wall to detect abnormalities, such as diverticula, stones, inflammation, voiding dysfunction, hematuria, anatomical dysfunction, irritable bladder syndrome, and tumors^1,2^.

Although cystoscopy has many benefits, patients may experience problems, such as pain and discomfort, because it is an invasive procedure. Pain tolerance may vary according to the type of cystoscope. Both flexible and rigid cystoscopes may be used during the procedure, and the use of a rigid cystoscope causes much more pain^3,4^. Reports that men experience more pain than women^5^ have raised the question of whether it is due to anatomical differences.

Despite its invasive nature and as recognized by international urological societies, it is only during cystoscopy that the patient's experience of pain, embarrassment, and anxiety can be evaluated accurately and in detail^6,7^ but the literature is limited. In the present study, we prospectively assessed the ­anxiety, pain, and embarrassment associated with cystoscopy and investigated the factors that may influence these to find possible ways to improve patient tolerance during the procedure.

## METHODS

### Study design and participants

The current research is a descriptive and cross-sectional study.

Data were collected from male patients who underwent planned cystoscopy in the Urology Operating Room of a ­hospital in Istanbul between October 2021 and 2022. Power ­analysis was conducted using the G*Power (3.1.9.2) program in order to determine the sample size. Based on the study,^1^ the sample size was determined at a 95% confidence level (=0.05 and two-way), 0.95 test power (1-), and Cohen's effect size d=0.26, resulting in a sample of 154 people. The study population consisted of all male patients scheduled for cystoscopy during the data collection dates, while the sample consisted of adult males who volunteered to participate in the study on the data collection date, were 18 years of age or older, and did not have hearing loss or neurological disorders that could affect the interview.

### Instruments

In this study, data were collected using a questionnaire prepared by the researchers, the Beck Anxiety Inventory (BAI), and the Numeric Rating Scale (NRS) for anxiety, embarassment, and pain. The questionnaire, developed by the researchers in accordance with the literature, contained questions on information on cystoscopy and the sociodemographic characteristics of patients. BAI measures the frequency of an ­individual's ­anxiety symptoms. It is a Likert-type self-assessment tool consisting of 21 items, each scored between 0 and 3. The total score ranges from 0 to 63. High total scores indicate a high level of ­anxiety experienced by the individual. It was ­developed by Beck et al.^8^; its Turkish validity and reliability were carried out by Ulusoy et al.^9^; the Cronbach's alpha value of the scale was found to be 0.93. Individuals’ total anxiety scores are interpreted as 0–17 low, 18–24 medium, and ≥25 high. In this study, the Cronbach's alpha value was found to be 0.90. The pain, embarrassment, and anxiety levels of the patients before and during the procedure were evaluated with a 0–10 NRS in the urology ­operating room. On the numerical pain scale, 0 means no pain, 1–3 indicates mild pain, 4–6 moderate pain, 7–9 severe pain, and 10 unbearable pain.

### Ethics statement

Before the study commencement, ethics committee approval was acquired from the Çukurova University Clinical Research Ethics Committee (approval number: 70/114, date: 10.09.2021), and necessary permissions were acquired from the hospital. The study adheres to the principles of the Helsinki Declaration. All subjects provided informed consent prior to data collection.

### Implementation of the study

To prevent the study results from being affected by procedural variables, cystoscopies were performed in a single hospital and were managed by a single physician and a single nurse. Patients were given standard information about the procedure in the ­waiting room. Before cystoscopy (T0), a descriptive form and BAI were applied. Cystoscopies were performed in the Trendelenburg position, starting with disinfection of the external genitalia and perineum. The urethra was lubricated with 2% lidocaine gel 5 min before cystoscopy insertion; a cystoscope (flexible or rigid; ≥17 Fr) appropriate for the purpose of the procedure was used. The researcher asked the patients to rate the levels of embarrassment and anxiety they experienced during perineal disinfection (T1) and cystoscope insertion (T2); and the levels of pain they experienced during cystoscope insertion (T2) and cystoscope removal (T3) with the NRS.

### Statistical analysis of data

The data obtained during the study were analyzed using Statistical Package for the Social Sciences (SPSS) (V.22). Number, percentage, and mean±standard deviation (SD) were used as descriptive statistical methods in the evaluation of the data. Kurtosis-Skewness values were analyzed to determine whether the research variables were normally distributed. In the related literature, the results of kurtosis and skewness values of the ­variables between ±1.5^10^ and ±2.0^11^ are accepted as normal distribution. It was determined that the variables showed normal distribution, so parametric methods were used to analyze the data. An independent groups t-test was used to compare quantitative continuous data between two independent groups. A dependent group t-test was used for the comparison of in-group measurements. A one-way analysis of variance (ANOVA) was used to compare quantitative continuous data between more than two independent groups. The least significant difference (LCD) test was used as a complementary post-hoc analysis to determine the differences after the ANOVA test.

## RESULTS

A total of 154 men were approached to participate. However, three patients who did not want to answer the questions asked during the procedure were excluded from the study, resulting in a final cohort size of 151 male patients. The mean age of the patients was 64.3±12.6 years, the body mass index (BMI) was 27.2±4.1 kg/m^2^, 86.1% were married, and 66.9% were primary school graduates. Just under half (45.7%) of the patients had a bladder tumor and underwent cystoscopy for routine follow-up, and overall, 64.9% had previous cystoscopy experience, 89.4% expected pain related to the procedure, and a rigid cystoscope was used in 60.9%. The mean T0_BAI_ of the patients was found to be 6.90±3.81. The mean of the measured parameters was found to be T1e=2.77±2.54 in the Trendelenburg position; T1a=1.79±1.23; T2e=2.32±2.30 during cystoscope insertion; T2a=1.94±1.28; T2p=1.44±0.75; and T3p=3.46±2.15 during cystoscope removal. The results showed that patients reported low levels of anxiety, pain, and embarrassment; however, there was a significant difference between the measurements on embarrassment and pain. Positive and statistically significant correlations were found between the BAI and all other variables. In particular, significant positive correlations were found between the T0_BAI_ and T3p (p<0.001), T2e (p<0.001), and T2a (p<0.001). In addition, there was a significant positive correlation between T1e and T2p (p=0.002) and between T1e and T1a (p<0.001) (Table 1).

**Table 1:** Correlation between Beck Anxiety Inventory, embarassment, anxiety, and pain mean of the patients.

Measures		T0_BAI_	T1e	T2e	T1a	T2a	T2p	T3p
T0_BAI_	R	1.000						
	P	0.000						
T1e	R	0.320**	1.000					
	P	0.000	0.000					
T2e	R	0.397**	0.875**	1.000				
	P	0.000	0.000	0.000				
T1a	R	0.370**	0.460**	0.444**	1.000			
	P	0.000	0.000	0.000	0.000			
T2a	R	0.635**	0.434**	0.470**	0.673**	1.000		
	P	0.000	0.000	0.000	0.000	0.000		
T2p	R	0.297**	0.252**	0.233**	0.524**	0.476**	1.000	
	P	0.000	0.002	0.004	0.000	0.000	0.000	
T3p	R	0.624**	0.390**	0.427**	0.290**	0.497**	0.248**	1.000
	P	0.000	0.000	0.000	0.000	0.000	0.002	0.000

** <0.01; Pearson correlation analysis. **T1: Trendelenburg position; T2: cystoscope insertion; T3: cystoscope removal.

In the regression model examining the effect of T0_BAI_ on embarrassment and pain scores of patients, T0_BAI_ significantly affected T1e (β=0.320, p<0.001) and T2e (β=0.397, p<0.001) and explained 9.6 and 15.2% of the variance, respectively. BAI also significantly affected the mean T2p (β=0.297, p<0.001) and T3p (β=0.624, p<0.001) and explained 8.2 and 38.6% of the variance, respectively. The results showed that there was a strong relationship between pre-procedure anxiety and post-procedure pain (Table 2).

**Table 2:** The effect of patients’ pre-procedure Beck Anxiety Inventory means on procedure embarrassment and pain means.

Independent variable	Unstandardized coefficients		Standardized coefficients	t	p	95%CI	
	B	SE	β			Alt	Top
Fixed	2.319	0.225		10.299	<0.001	1.874	2.764
Beck Anxiety Score	0.118	0.029	0.320	4.119	<0.001	0.061	0.174
*Dependent variable=T1e, r=0.320; R^2^=0.096; F=16.963; p=0.000; Durbin Watson Value=1.943							
Fixed	1.788	0.199		8.964	<0.001	1.394	2.182
Beck Anxiety Score	0.134	0.025	0.397	5.280	<0.001	0.084	0.184
*Dependent variable=T2e, r=0.397; R^2^=0.152; F=27.880; p=0.000; Durbin Watson Value=2.078							
Fixed	0.512	0.129		3.982	<0.001	0.258	0.766
Beck Anxiety Score	0.062	0.016	0.297	3.794	<0.001	0.030	0.094
*Dependent variable=T2p, r=0.297; R^2^=0.082; F=14.397; p=0.000; Durbin Watson Value=1.768							
Fixed	2.722	0.157		17.342	<0.001	2.412	3.032
Beck Anxiety Score	0.195	0.020	0.624	9.756	<0.001	0.155	0.234
*Dependent variable=T3p, r=0.624; R^2^=0.386; F=95.180; p<0.001; Durbin Watson Value=1.842							

* T1e: Embarrassment in the Trendelenburg position. *T2e: Embarrassment when inserting a cystoscope. *T2p: Pain when inserting the cystoscope. *T3p: Pain when removing the cystoscope. CI: confidence interval; SE: standard error.

Changes in research variables are given in Table 3. T0_BAI_ and T2a scores of patients with normal BMI are higher than the others (p=0.002; p=0.008). Hematuria (p=0.000), having a second cystoscopy (p=0.008) is effective in high pain average; and using a Flexible (≥17 Fr) cystoscope is effective in low pain average (Table 3).

**Table 3:** Differentiation of anxiety, embarrassment, and pain means of patients according to demographic characteristics.

		n	T0_BAI_	T1e	T2e	T1_a_	T2_a_	T2p	T3p
			Mean±SD	Mean±SD	Mean±SD	Mean±SD	Mean±SD	Mean±SD	Mean±SD
BMI	Normal	46	9.830±6.650	3.370±2.594	2.780±2.356	1.951±1.720	2.149±1.960	1.697±0.910	3.870±2.500
	Overweight	75	5.228±2.930	2.611±2.470	2.427±2.120	1.695±1.070	1.889±1.120	1.236±0.650	3.470±2.088
	Obese	30	2.684±1.630	2.600±2.207	2.000±1.965	1.676±0.870	1.450±0.630	1.530±0.730	2.830±1.577
F			6.426	1.896	1.470	2.684	4.933	0.460	2.134
p			**0.002** *	0.154	0.233	0.072	**0.008** *	0.632	0.122
Post-hoc			1>2, 1>3				1>2, 1>3		
Reason for cystoscopy	Abduction Impulse	10	2.600±2.757	3.500±2.635	2.800±1.932	1.300±1.567	1.947±1.700	1.350±0.600	2.600±2.547
	Retention	25	5.835±3.280	3.200±2.533	2.480±2.201	1.675±0.840	1.528±0.800	1.044±0.560	3.640±1.655
	Hematuria	23	8.508±3.130	3.130±2.380	2.520±2.108	2.172±2.090	2.020±1.520	2.066 ±1.780	3.300±2.285
	Routine Monitoring	69	7.253±4.330	2. 653±2.410	2. 471±2.120	1.654±1.000	2.007±1.130	0.953±0.280	3.260±2.048
	Stent removal	24	6.737±4.00	2.710±2.386	2.502 ±2.210	1.840±1.420	2.064±1.790	1. 663±1.380	4.380±2.464
F			0.268	0.852	0.316	2.014	1.105	7.049	1.737
p			0.898	0.495	0.867	0.095	0.357	**0.000** *	0.145
Post-hoc								3>1, 3>2, 3>4, 5>2, 5>4	
Number of cystoscopy	First	53	7.873±4.550	3.080±2.409	2.750±2.401	1.758±1.150	1.889±1.170	1.464±0.830	3.890±2.309
	Second	18	9.083±4.830	3.220±2.713	2.560±2.202	2.279±1.610	2.437±1.940	2.191±1.720	3.890±1.711
	Third	19	3.025±2.530	2.859±2.790	2. 353±2.260	1.686±1.210	2.065±1.470	1.003±0.320	2.890±2.283
	>3	61	6.142±3.260	2. 517±2.360	2.260±1.840	1.727±1.180	1.799±1.110	1.134±0.520	3.150±2.040
F			0.676	0.968	1.572	0.317	0.964	4.084	1.826
p			0.568	0.409	0.199	0.813	0.411	**0.008** *	0.145
Post-hoc								2>1, 2>3, 2>4	
Cystoscope type and number	Rigid (17 Fr)	92	4.460±7.616	3.090±2.646	2.500±2.411	1.837±1.250	2.134±1.340	1.279±0.540	3.800±2.165
	Rigid (>17 Fr)	21	4.290±6.849	2.373±2.330	2. 406±2. 100	1.729±1.240	1.806±1.480	1.430±1.399	4.050±2.312
	≥Flexible (17 Fr)	38	1.970±4.541	2.240±2.318	2.071±1.920	1.763±1.160	1.515±1.030	1.727±0.870	2.320±1.596
F			1.817	1.876	0.922	0.036	0.465	3.504	8.007
p			0.166	0.157	0.400	0.965	0.629	**0.033** *	**0.000** *
Post-hoc								2>1	1>3, 2>3

F: anova test; t: independent groups t-test; post-hoc: Tukey, LSD. SD: standard deviation; BMI: body mass index.

* Bold values represent statistically significant differences (p<0.05).

## DISCUSSION

Cystoscopy is an everyday procedure used for routine monitoring of bladder cancer and evaluation of patients with lower urinary tract symptoms, such as hematuria. The invasive nature of this procedure may cause significant anxiety, pain, and embarrassment in patients. More than half of patients who experience cystoscopy are negatively affected psychologically and ­physiologically^12,13^. Male and young patients are more sensitive to feelings of anxiety and embarrassment during cystoscopy, and demographic differences affect these parameters in patient experience^1^.

Although there are practices aiming to reduce patient ­anxiety, embarrassment, and pain during cystoscopy, they have not been evaluated together, and at which step these variables differ has not been investigated previously^14,15^. In the research, we present a prospective systematic evaluation of adult male patients’ experience of cystoscopy. It shows that patients’ tolerance to the cystoscopy procedure was good, with positive correlations between anxiety, pain, and embarrassment generally. Anxiety, pain, and embarrassment reported before and during invasive urological procedures were highly correlated with each other and parallel with previous research^16,17^.

Embarrassment, caused by social stigmatization during urological interventions, increases anxiety associated with cystoscopy. Embarrassment and stigmatization may prevent open communication between patients and health professionals, limit the care, cause isolation, and delay medical consultations^18,19^. Addressing these emotional barriers is important to improve patients’ experiences with cystoscopy. The results showed that there was a difference between the embarrassment and pain measurements. The Trendelenburg position, exposure of the meatus due to the nature of the procedure, and the ­inability to maintain confidentiality appear to have had an effect on the embarrassment score. The application of lubricating gel-lidocaine to the narrow meatus of a man, especially during cystoscope removal, may not have provided adequate analgesia. However, the patient's anxiety before the procedure was a determinant of pain level.

In urological endoscopy, due to privacy and individual ­reservations, patients cannot question the procedure steps, cannot understand them, and experience anxiety^20^. It is reported in the literature that this situation can be reduced with visual information, interpersonal, and communication skills of care professionals^21^.

It appears that anxiety has a strong effect on the patient's sense of pain and embarrassment. The results, which are similar to the literature^16,21^, anxiety is an important symptom that should be eliminated before the procedure.

The symptoms related to urological procedures should be evaluated with a holistic approach. We believe that the procedure stages, such as position, cystoscope selection, and natural manipulation of the cystoscope, may be decisive in affecting the perceptions of the symptoms.

We could not interpret the high BMI on the low anxiety levels of individuals; however, patients with hematuria may have felt more pain due to degeneration of the urinary tract. Flexible cystoscopies are less preferred due to high cost, limited view, and technical difficulty^16^. In this study, it was found that cystoscopy type <17Fr is effective on pain, while cystoscope size is effective in those >17Fr. Standard information before the procedure is now routine; it can reduce pain and anxiety^17,22^. Since patients are awake during cystoscopy, communication should be maintained throughout the procedure^23,24^. In the study where we revealed the effects of the procedure steps, it was thought that moderate pain and anxiety could be a natural result of standard information before the procedure and communication established for measurement during the procedure.

## CONCLUSION AND RECOMMENDATIONS

This study highlighted the strong correlations between anxiety, pain, and embarrassment reported by male patients during the cystoscopy procedure. The results suggest that the procedure steps affect these parameters to different degrees. To reduce embarrassment during cystoscopy, the position adopted by the patient can be determined according to the patient's preference. Flexible cystoscopes may be preferred for reducing pain. In addition, the patient's pain should be repeatedly assessed when the cystoscope is removed, which may help to alleviate pain from the exit manipulation.

## ETHICAL CONSIDERATIONS

Before the study commencement, ethics committee approval was acquired from the Çukurova University Clinical Research Ethics Committee (approval number: 70/114, date: 10.09.2021), and necessary permissions were acquired from the hospital. The study adheres to the principles of the Helsinki Declaration. All subjects provided informed consent prior to data collection.

## Data Availability

The datasets generated and/or analyzed during the current study are available from the corresponding author upon reasonable request.
